# De-regulation of the sonic hedgehog pathway in the InsGas mouse model of gastric carcinogenesis

**DOI:** 10.1038/sj.bjc.6603782

**Published:** 2007-05-15

**Authors:** M El-Zaatari, A Tobias, A M Grabowska, R Kumari, P J Scotting, P Kaye, J Atherton, P A Clarke, D G Powe, S A Watson

**Affiliations:** 1 Division of Pre-Clinical Oncology, University of Nottingham, Nottingham, UK; 2 Institute of Genetics, University of Nottingham, Nottingham, UK; 3 Division of Pathology, University of Nottingham, Nottingham, UK; 4 Wolfson Digestive Diseases Centre, University of Nottingham, Nottingham, UK

**Keywords:** sonic hedgehog, CCK-2R, pre-malignant, metaplastic, InsGas, *H. felis*

## Abstract

This study investigated sonic hedgehog (Shh) signalling in gastric metaplasia in the insulin-gastrin (InsGas) hypergastrinaemic mouse +/− *Helicobacter felis* (*H. felis*) infection. Sonic hedgehog gene and protein expression was reduced in pre-metaplastic lesions from non-infected mice (90% gene reduction, *P*<0.01) compared to normal mucosa. Sonic hedgehog was reactivated in gastric metaplasia of *H. felis*-infected mice (3.5-fold increase, *P*<0.01) compared to pre-metaplastic lesions. Additionally, the Shh target gene, glioma-associated oncogene (Gli)-1, was significantly reduced in the gastric glands of InsGas mice (75% reduction, *P*<0.05) and reactivated with *H. felis* infection (*P*<0.05, base of glands, *P*<0.01 stroma of metaplastic glands). The ability of *H. felis* to activate the Shh pathway was investigated by measuring the effect of target cytokine, interleukin-8 (IL-8), on Shh expression in AGS and MGLVA1 cells, which was shown to induce Shh expression at physiological concentrations. *H. felis* induced the expression of NF-*κ*B in inflammatory infiltrates *in vivo*, and the expression of the IL-8 mouse homologue, protein KC, in inflammatory infiltrates and metaplastic lesions. Sonic hedgehog pathway reactivation was paralleled with an increase in proliferation of metaplastic lesions (15.75 *vs* 4.39% in infected *vs* non-infected mice, respectively, *P*<0.001). Furthermore, Shh overexpression increased the growth rate of the gastric cancer cell line, AGS. The antiapoptotic protein, bcl-2, was expressed in the stroma of infected mice, along with a second Shh target gene, *patched-1* (*P*=0.0001, stroma of metaplastic gland). This study provides evidence suggesting reactivation of Shh signalling from pre-metaplastic to advanced metaplastic lesions of the stomach and outlines the importance of the Shh pathway as a potential chemoprophylactic target for gastric carcinogenesis.

Gastric cancer is one of the most common forms of cancer worldwide with an incidence of 934 000 new cases per year (Parkin *et al*, 2005). *Helicobacter pylori* (*H. pylori*) is a major risk factor linked to gastric carcinogenesis and has been classified by the International Agency for Research on Cancer (IARC) as a carcinogenic bacterium in humans (IARC, 1994).

A number of studies have shown an increase in sonic hedgehog (Shh) production in human gastric tumour biopsies (Ma *et al*, 2005; Ohta *et al*, 2005), and high expression of the Shh pathway in gastric tumour cell lines (Berman *et al*, 2003). The importance of the overexpressed Shh pathway observed in gastric cancer has been demonstrated by the ability of Shh pathway antagonists to suppress growth of gastrointestinal (GI) tumour cell lines and xenografts (Berman *et al*, 2003; Ma *et al*, 2005; Ohta *et al*, 2005). However, Shh expression was shown to be reduced in pre-malignant lesions, atrophic gastritis and intestinal metaplasia (van den Brink *et al*, 2002; Shiotani *et al*, 2005) and during acute infection with *H. pylori* in Mongolian gerbils (Suzuki *et al*, 2005). This reduced expression may be partially explained by the loss of Shh pathway-expressing cell types during gastric atrophy and early metaplasia.

In this study, the insulin-gastrin (InsGas) transgenic mouse model of gastric carcinogenesis has been used to study Shh pathway expression during this process. *Helicobacter felis* (*H. felis*)-infection of InsGas mice enhanced transformation of the gastric mucosa (Wang *et al*, 2000), and so we aimed to compare Shh pathway activation between pre-metaplastic and pseudopyloric metaplastic lesions in the non-infected and *H. felis*-infected InsGas models, respectively, focussing on Shh target genes, *Patched-1* (*Ptch-1*) and *glioma-associated oncogene* (*Gli*)-*1* and a factor, which regulates the pathway but which is not a transcriptional target, *Gli-3*.

We hypothesised that the discrepancy, reported in the literature, in Shh expression between pre-malignant lesions and cancer may be in response to an elevated inflammatory response, which may be able to trigger Shh expression.

## MATERIALS AND METHODS

### Animals

FVB and InsGas mice were bred, under sterile conditions, within the Academic Unit of Cancer Studies, under Home Office Project License No. 40/2323. Insulin-gastrin mice either remained non-infected, or were infected with *H. felis* (dosed orally with either 1 × 10^8^ colony-forming units of *H. felis* in brucella broth or equivalent volumes of broth alone on days 1, 3 and 5). Mice were maintained for 10 months and at termination were injected with bromodeoxyuridine (BrdU) 1 h before sacrifice. Stomach tissue was dissected out and frozen in optimal cutting temperature compound (Bayer plc, Berkshire, UK), by immersion into liquefied Richard-Allan Cytocool II (Apogent, Ipswich, UK) on dry ice. Frozen blocks and cryostat sections were stored at −80°C.

### Cell culture

The human gastric adenocarcinoma cell lines AGS (ECACC, Wiltshire, UK) and MGLVA1 (Division of Pre-clinical Oncology, Nottingham University, (Watson *et al*, 1990)) were routinely cultured in RPMI 1640 culture medium (Gibco, Paisley, UK) containing 10% (v/v) heat-inactivated foetal bovine serum (Sigma, Poole, UK) at 37°C in 5% CO_2_ and humidified conditions. In some experiments, interleukin-8 (IL-8) (R&D, Abingdon, UK) was added at concentrations of 0.1–10 nM to cells, which had been plated at 2.5 × 10^4^ cells per well in 24-well plates and serum-starved over-night.

### Navigated laser capture microdissection

Frozen sections (10 *μ*m) were mounted on PALM PEN membrane slides for microdissection using a PALM microdissector (PALM Microlaser Technologies, Bernried, Germany). Sections were air-dried, fixed in pre-chilled (−30°C) 70% RNase-free ethanol at room temperature, counterstained with RNase-free toluene blue (Sigma, Poole, UK), and washed in RNase-free PBS. The slides were then baked at 40^o^C for 40 min before microdissection. Pre-metaplastic and metaplastic lesions were micro-dissected following characterisation by a pathologist.

### RNA extraction and cDNA synthesis

Dissected tissue was collected in 350 *μ*l buffer RLT containing *β*-mercaptoethanol from an RNEasy microkit (Qiagen). RNA was reverse transcribed using Superscript III reverse transcriptase (Invitrogen, Paisley, UK) according to the manufacturer's instructions.

### Quantitative polymerase chain reaction

Gene expression was quantified using reagents from the quantitative polymerase chain reaction (qPCR) Core kit for SYBR Green I (Eurogentec, Romsey, UK). Primers were designed using Primer Express version 2 (Applied Biosystems, Foster City, CA, USA, Table 1), and PCR assays carried out on a 7500 Sequence Detection System (PE Biosystems, Warrington, UK). Primer efficiency curves were performed, and results expressed relative to the housekeeping gene (*β-actin* for *Shh*, *Gli-1*, and *Gli-3*, and *RPL13a* for *Ptch-1*) using the 2^−ΔΔCT^ values and relative quantitation (Livak and Schmittgen, 2001) according to the following equation:

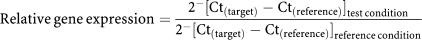


### Antibodies

Polyclonal antibodies directed against Shh (N-19, 4 *μ*g/ml), protein KC (8 *μ*g/ml) and CCK-2/gastrin receptor (CCK-2R) (S-20, 12 *μ*g/ml) were obtained from Santa Cruz (Autogen Bioclear, Wiltshire, UK). Monoclonal antibody directed specifically against the active form of the p65 subunit of NF-*κ*B was obtained from Chemicon (Harrow, UK). Monoclonal antibodies to BrdU (M0744 5.56 *μ*g/ml) and bcl-2 (M0887 5.6 *μ*g/ml) were obtained from Dako (Ely, UK) and binding of primary antibodies was detected using HRP-conjugated secondary antibodies (Dako).

### Immunohistochemistry

Sonic hedgehog and CCK-2R staining was performed on frozen sections, whereas bcl-2, BrdU, KC and NF-*κ*B staining was performed on paraffin sections. Paraffin sections were de-waxed in xylene, washed in 100% ethanol and underwent antigen retrieval in 10 mM, pH 6.0 citrate buffer. Frozen sections were air-dried and fixed in 4% paraformaldehyde. Sections were washed in PBS and blocked for endogenous peroxidase activity (1.2% H_2_O_2_ for Shh N-19 and KC antibodies, and 0.06% H_2_O_2_ for CCK-2R). Sections were then washed in PBS, blocked in 10% rabbit serum, and incubated with primary antibody. For negative controls, concentration- and species-matched serum or IgG were applied. The sections were washed in PBS, incubated with secondary antibody, and washed again in PBS. Staining was developed using liquid diaminobenzidine (DAB, Dako). Bromodeoxyuridine labelling and active NF-*κ*B staining was performed using the Ark-labelling kit (Dako).

### Construction of Shh-expressing plasmid and stable transfection into AGS cells

pJT4/Shh, containing the chick *Shh* insert, was kindly provided by Professor Hiroshi Sasaki. The R-U5 sequence, splicing/intron sequence, and Shh insert were excised by *Xho*I digestion and ligated into the *Xho*I site of pcDNA3.1(+) (Invitrogen), which was confirmed by sequencing. For vector control, pcDNA3.1(+) was transfected into AGS cells. Transfection was carried out using Lipofectamine 2000 (Invitrogen), and high-expressor colonies chosen for cell-growth analysis following single-cell cloning. Cell growth analysis was performed using 3-(4, 5-dimethylthiazolyl-2)-2, 5-diphenyltetrazolium bromide (MTT, Sigma).

### Statistical analysis

Gene expression was analysed by the Mann–Whitney *U* non-parametric statistical test, proliferation assessed (BrdU uptake) by the Student's *t*-test and MTT uptake by a one-way analysis of variance.

## RESULTS

### Expression of Shh is lost in pre-malignant lesions and reactivated in advanced lesions

**Figure 1:**
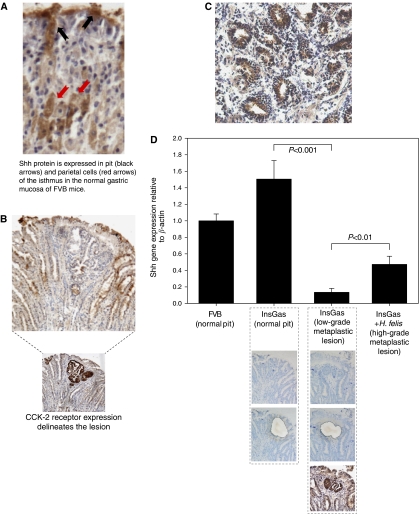
Expression of Shh in normal and metaplastic gastric mucosa in the InsGas mouse. (**A**) Shh protein is expressed in pit and parietal cells of the isthmus in the normal gastric mucosa of FVB mice. (**B**) Loss of Shh protein expression in pre-metaplastic gastric lesions of non-infected InsGas mice (lesion delineated by CCK-2 receptor expression). (**C**) Reactivation of Shh protein expression in pseudopyloric metaplastic lesions from *H. felis*-infected InsGas mice. (**D**) CCK-2/gastrin receptor protein expression delineated the lesion in non-infected InsGas, and was used to navigate the microdissection of the lesion for RNA analysis. Tissue from the pit region of normal adjacent glands in the same section was used as a control. RT-PCR analysis of the microdissectates shows a significant 90% reduction in Shh gene expression in pre-metaplastic lesions from non-infected InsGas mice (*P*<0.001, Mann–Whitney *U*-test), but a 3.5-fold re-activation in pseudopyloric metaplasia from *H. felis*-infected InsGas (*P*<0.01). Error bars=95% confidence interval.

*Sonic hedgehog* protein was localised to both pit and parietal cells of the normal gastric mucosa (Figure 1A), but was lost in pre-metaplastic lesions of non-infected InsGas mice (as defined by a pathologist and delineated by gastrin/CCK-2R expression) compared to normal adjacent glands (Figure 1B). Pseudopyloric metaplasia in *H. felis*-infected mice exhibited reactivation of Shh protein expression (Figure 1C). The loss of Shh protein was corroborated at the gene level by laser capture microdissection (LCM) of pre-metaplastic lesions (Figure 1D). *Sonic hedgehog* gene expression was downregulated by greater than 90% in pre-metaplastic lesions from non-infected mice compared to normal adjacent tissue from the same section (*P*<0.001, Figure 1D), but was partially recovered in metaplastic lesions of *H. felis*-infected mice (3.5-fold increase, *P*<0.01, Figure 1D).

### *H. felis* infection may enhance Shh expression via the upregulation of mouse IL-8 homologue, protein KC, in infiltrating inflammatory cells

**Figure 2:**
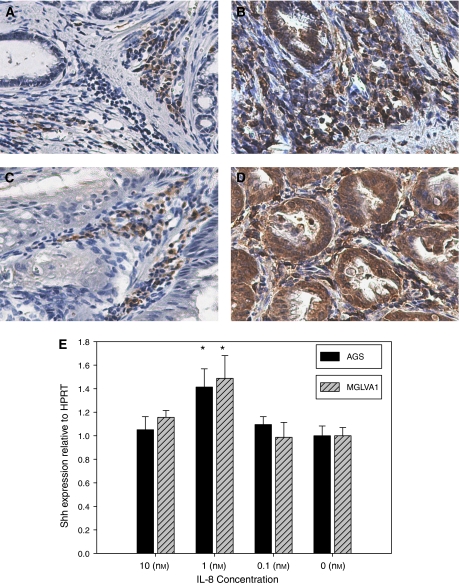
Upregulation of NF-*κ*B in *H. felis* infection. Expression of NF-*κ*B (**A**, **C**) and IL-8 (**B**, **D**) in infiltrating cells at the base of gastric glands (**A**, **B**) and the stroma surrounding pre-metaplastic lesions (**C**, **D**) during *H. felis* infection, IL-8 staining is also present in pre-metaplastic lesions (**D**), magnification × 20. Sonic hedgehog gene expression was upregulated in AGS and MGLVA1 gastric cell lines (**E**) following treatment with 1 nM IL-8 (^*^P<0.0001).

*H. felis* infection increased the expression of NF-*κ*B and the IL-8 homologue, KC, in infiltrating cells at the base of gastric glands (Figure 2A and B), and in the stroma surrounding pre-metaplastic lesions (Figure 2C and D). KC was also expressed in pre-metaplastic lesions (Figure 2D) where Shh was detected (Figure 1C). No staining was seen in these areas in the negative controls. Interleukin-8 treatment of AGS and MGLVA1 cells significantly increased Shh gene expression at physiological levels (nM) (Figure 2E).

### Expression of Gli-transcription factors during gastric carcinogenesis

**Figure 3:**
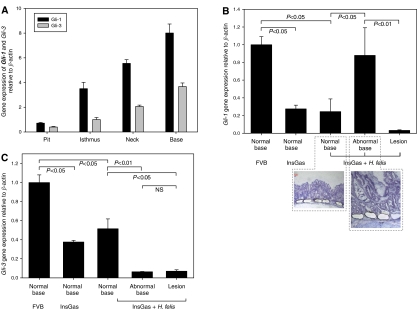
Gli-transcription factor gene expression in metaplastic progression in the InsGas mouse. (**A**) Relative expression of *Gli-1* and *Gli-3* in the normal mucosa showing highest expression of each factor at the base of gastric glands. (**B**) *Gli-1* gene expression is reduced in the normal base of InsGas mice (±*H. felis* infection) but reactivated in the base region associated with pseudopyloric metaplasia (*P*<0.05, Mann–Whitney *U*-test). (**C**) *Gli-3* expression is reduced with increasing malignant potential and not reactivated in pseudopyloric metaplasia. Error Bars=95% confidence limits, NS=nonsignificant.

The highest expression of Gli-1 and Gli-3 was detected in the base region of normal murine gastric mucosa (Figure 3A). *Gli-1* and *Gli-3* gene expression was measured in microdissected base tissue from gastric units of InsGas mice with/without *H. felis* infection (Figure 3B and C). Target *Gli-1* expression was significantly reduced in the base of normal gastric units in both non-infected (73% inhibition, *P*<0.05) and *H. felis*-infected InsGas mice (75% inhibition, *P*<0.05) (Figure 3B). Expression of *Gli-1* was reactivated in the base region of metaplastic lesions in infected mice, compared with adjacent normal regions (3.3-fold increase, *P*<0.05, Figure 3B).

The expression of Gli-3 RNA was also reduced with increasing malignant potential (normal base of gastric units in both non-infected and infected InsGas mice, *P*<0.05 when compared to normal base from wild-type FVB mice, Figure 3C). Expression was not reactivated in the base region of abnormal gastric glands or metaplastic lesions in *H. felis*-infected InsGas mice (Figure 3C).

### Enhanced cell growth and antiapoptotic activity in advanced lesions may be attributed to paracrine Shh signalling

**Figure 4:**
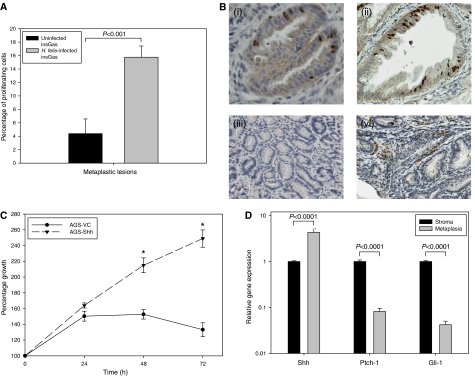
Proliferation rate and bcl-2 expression in metaplastic glands of the InsGas mouse and hedgehog pathway gene expression in associated stroma. (**A**) Proliferation rate was increased significantly in pre-metaplastic lesions of *H. felis*-infected compared to non-infected InsGas mice (*P*<0.001, Student's *t*-test). (**B**) Proliferative nuclei and bcl-2 expression in *H. felis*-infected (ii and iv) compared to non-infected (i and iii) InsGas mice, respectively. Magnification × 20. (**C**) Sonic hedgehog-transfected AGS cells exhibit an increased growth rate compared with vector control-transfected cells (*P*<0.0001, one-way analysis of variance). (**D**) Expression of Shh, Ptch-1 and Gli-1 in pseudopyloric metaplastic lesions compared to the surrounding stroma. Error bars=95% confidence intervals.

The percentage of proliferating cells was significantly higher in pseudopyloric metaplasia in *H. felis*-infected when compared to pre-metaplastic lesions in non-infected InsGas mice (15.76 *vs* 4.39%, *P*<0.001, Figure 4A and visually depicted in B). In accordance with this, Shh overexpression in the human gastric adenocarcinoma cell line, AGS, promoted growth significantly compared with vector control-transfected cells in serum free medium (Figure 4C, *P*<0.0001).

Apoptotic potential was determined by examining bcl-2 expression, a known down-stream, antiapoptotic factor associated with hedgehog signalling (Bastida *et al*, 2004) and expression was confirmed in the stroma associated with metaplastic lesions in infected but not non-infected mice (Figure 4B). The stroma was subjected to LCM and it was shown that whereas Shh was not expressed, Shh target genes, *Ptch-1* and *Gli-1*, were co-expressed in the stroma surrounding Shh-expressing lesions in *H. felis*-infected InsGas mice (Figure 4D).

## DISCUSSION

This study has confirmed that Shh expression and signalling is reduced in early pre-metaplastic lesions in non-infected InsGas mice as shown previously (van den Brink *et al*, 2002; Shiotani *et al*, 2005). However, in pseudopyloric metaplastic lesions arising in *H. felis*-infected mice, Shh is re-expressed, together with distinct expression of the down-stream target gene, *Gli-1*, in the base and associated stroma, suggesting paracrine activity.

Previous studies have confirmed that Shh and its target gene *Ptch-1* are upregulated in inflamed tissues of the GI tract (Nielsen *et al*, 2004) with human gastritic epithelium (including that associated with *H. pylori* infection) shown to strongly express Shh. This may be a direct effect of infection, as *Helicobacter* activates NF-*κ*B via chronic inflammation and the activation of interleukins, such as IL-4 (Lee *et al*, 2005), or in response to lipopolysaccharides (Hynes *et al*, 2004) and NF-*κ*B in turn is able to activate Shh (Nakashima *et al*, 2006). We have shown in this study that one mechanism may be via the activation of NF-*κ*B and its transcriptional target, IL-8 (Aihara *et al*, 1997; Chu *et al*, 2003), in infiltrating inflammatory cells surrounding the lesions, as IL-8 was shown to induce Shh gene expression in gastric adenocarcinoma cells.

Functional Shh signalling was confirmed, as the hedgehog target, *Gli-1*, was re-expressed in the base and stroma of pseudopyloric metaplastic lesions in *H. felis*-infected mice, in contrast to *Gli-3*, which is not a transcriptional target of the Shh pathway, and which maintained low expression.

Gli-1 is known to induce expression of proliferative genes such as cyclin D2 (Yoon *et al*, 2002), FOXM1 and the ras-ERK pathway (Xie *et al*, 2001; Teh *et al*, 2002) and to reduce expression of cell cycle repressor p21/CIP1 in gastric carcinoma cell lines (Ohta *et al*, 2005). Gli-1 was also shown to potentiate the malignant phenotype via its antiapoptotic activity in gastric tumour cells (Ma *et al*, 2005). In the present study, there was a higher rate of proliferation and antiapoptotic activity in metaplastic compared with in pre-metaplastic lesions in non-infected mice and Shh overexpression increased the growth rate of a gastric cell line.

In contrast to Gli-1, the proapoptotic factor, Gli-3, which is not a transcriptional target of the hedgehog pathway, was not reactivated in metaplastic glands. This may therefore result in a switch to an antiapoptotic, proproliferative phenotype as Gli-3 repressor activity is associated with an increased level of proapoptotic BMP-4 (Bastida *et al*, 2004). Furthermore, in reduced Shh signalling, Ptch-1 can induce an apoptotic signal through Gli-3 (Kalderon, 2004). Gli-3 also inhibits Gli-1's ability to upregulate bcl-2 transcription in basal cell carcinoma (Bastida *et al*, 2004).

Interestingly, Ptch-1 and Gli-1, both transcriptional targets of hedgehog signalling were overexpressed in the stroma surrounding metaplastic lesions in *H. felis* infected but not in non-infected mice and paralleled an upregulation of bcl-2 expression. Although the cell type(s) responsible for expression were not confirmed in the present study, Ptch-1 is known to be expressed by infiltrating immune cells (Stewart *et al*, 2002), which could be responsible for this expression. Furthermore, enhanced bcl-2 expression may be as a result of Gli-1 re-expression as confirmed in gastric carcinoma cells (Ohta *et al*, 2005).

Finally, we detected Shh expression in pit and parietal cells of the gastric mucosa, whereas a recent study has detected Shh only in parietal cells (Fukaya *et al*, 2006). Sonic hedgehog has previously been reported to be absent in pit cells in one study (van den Brink *et al*, 2001), whereas another study detected Shh expression in the pit region of Mongolian gerbils by *in situ* hybridisation (Suzuki *et al*, 2005). The expression of Shh in the pit may differ between FVB mice utilised in this study and mouse strains utilised in other studies (van den Brink *et al*, 2001; Fukaya *et al*, 2006). We have, however, confirmed *Shh* RNA expression by LCM, in which high expression of *Shh* was detected in the pit region of FVB mice. The discrepancy may also be attributed to the specificity of the antibodies in question, which may crossreact with Ihh (Van Den Brink and Peppelenbosch, 2006). The expression of *Gli-1* was also detected in this study by LCM throughout the gastric gland, with highest expression in base cells. This correlates with Gli-1 expression detected in a recent study (Yanai *et al*, 2007), but disagrees with observations made by Fukaya *et al* (2006) that Gli-1 expression is absent in the isthmus/neck region. The pattern of *Gli-1* expression in this study was obtained consistently in different samples using quantitative RT-PCR and primers that span the exon–exon junctions to avoid genomic contamination. Samples also underwent DNase treatment, and primers were designed not to overlap with homologous genes, *Gli-2* and *Gli-3*. Therefore, the reason for the discrepancy remains unclear, but we speculate that this may be attributed to the sensitivity of detection of gene expression using quantitative or classical PCR, the former being normalised against housekeeping gene expression, whereas the latter is dependent on the relative amount of cDNA loaded into the reaction.

We propose that Shh expression is downregulated in pre-metaplastic gastric lesions in the InsGas mouse, with an associated decrease in *Gli-1* expression. In pseudopyloric metaplasia, as a result of *H. felis* infection, Shh is reactivated and may signal, in a paracrine manner, to activate Gli-1 in the stroma and the base regions. Reactivation of Shh may be mediated by the induction of inflammatory cytokines in response to *H. felis* infection. This study suggests that the Shh pathway is reactivated in pseudopyloric metaplasia, and may contribute to gastric carcinogenesis and therefore may be an appropriate chemo-prophylactic target.

## Figures and Tables

**Table 1:** Primer sequences

**Primer**	**Sequence**
Mouse *Shh*	Forward: 5′-AGGAACTCACCCCCAATTACAAC-3′
	Reverse: 5′-AGAGATGGCCAAGGCATTTAACT-3′
Mouse *Ptch-1*	Forward: 5′-GTGATTGTGGAAGCCACAGAAA-3′
	Reverse: 5′-TGTCTGGAGTCCGGATGGA-3′
Mouse *Gli-1*	Forward: 5′-GCTGGAGGTCTGCGTGGTA-3′
	Reverse: 5′-GGTGGAGTCATTGGATTGAACA-3′
Mouse *Gli-3*	Forward: 5′-AGGTCAGCTCTGGCCCTTCT-3′
	Reverse: 5′-AGGGTCACCAGTGCTGCTCA-3′
Mouse *RPL13A*	Forward: 5′-GCGCCTCAAGGTGTTGGAT-3′
	Reverse: 5′-GAGCAGCAGGGACCACCAT-3′
Mouse *β-actin*	Forward: 5′-GCTTCTTTGCAGCTCCTTCGT-3′
	Reverse: 5′-CCAGCGCAGCGATATCG-3′
